# Clinical *Nocardia* species: Identification, clinical characteristics, and antimicrobial susceptibility in Shandong, China

**DOI:** 10.17305/bjbms.2020.4764

**Published:** 2020-11

**Authors:** Shu-Hua Lu, Zhen-Wen Qian, Pei-Pei Mou, Lian Xie

**Affiliations:** 1 Department of Clinical Laboratory, Affiliated Hospital of Jining Medical University, Jining, China; 2 Department of Clinical Laboratory, Shengli Oilfield Central Hospital, Dongying, China

**Keywords:** *Nocardia*, *Nocardia* infection, nocardiosis, antimicrobial susceptibility, mass spectrometry, identification

## Abstract

*Nocardia* is a pathogen responsible for a variety of clinical infections. Here, we aimed to investigate the species distribution, clinical manifestations, and antimicrobial susceptibility of *Nocardia* species over 3 years in two tertiary general hospitals in China. In this retrospective study, a total of 27 *Nocardia* species were isolated from 27 individuals between January 2017 and December 2019. *Nocardia* isolates were identified to species level by mass spectrometry and 16S rRNA PCR sequencing. Clinical data were collected from medical records. Antimicrobial susceptibility was determined by the standard Broth microdilution method. The 27 patients with *Nocardia* infection included 12 males and 15 females with a mean age of 60.11 years. Among 27 *Nocardia* isolates, 7 species were identified, with the most common species being *Nocardia otitidiscaviarum* (40.7%). The antimicrobial susceptibility profiles varied between different *Nocardia* species. Notably, all *Nocardia* isolates were linezolid susceptible. The majority of *Nocardia* isolates were collected from a department of respiratory medicine (55.56%) and sputum specimen (44.44%). Pulmonary region was the most involved body site (70.37%) followed by skin (7.4%) and pleural cavity (7.4%). Most patients with *Nocardia* infection needed combination antibiotic therapy. Two deaths were reported during the treatment period and 24 patients achieved improvement after antibiotic therapy. The clinical manifestations of *Nocardia* infection and antimicrobial susceptibility profiles varied with diverse *Nocardia* species. Thus, the accurate identification of these species is crucial for the diagnosis and the selection of antibiotic treatment.

## INTRODUCTION

*Nocardia* species, an aerobic actinomycete, exist in a wide range of environments around the world [1]. Currently, only a small proportion of described *Nocardia* species are known to be human pathogens that affect patients [2]. *Nocardia* species are facultative intracellular pathogens, which are capable of causing either a localized or disseminated infection both in immunocompetent and immunocompromised hosts [3]. In recent decades, an increased number of *Nocardia* infection cases have been reported worldwide [4]. *Nocardia* infection is characterized by a variety of clinical manifestations with the involvement of the lungs, central nervous system, skin, and other organs. More seriously, *Nocardia* can cause severe, life-threatening disseminated infections, such as osteomyelitis and nocardial sepsis [5,6]. Early recognition and effective therapy are imperative to achieve successful outcomes. Thus, elaboration of the molecular characteristics, infection, and clinical manifestations of *Nocardia* species is necessary for timely detection and diagnosis.

Despite numerous *Nocardia* species have been characterized both phenotypically and genotypically within the genus, the genotype remains heterogeneous and continues to evolve [7]. In addition, the genus of *Nocardia* is rapidly expanding and the species distribution varies with different geographical locations [8]. Reports about *Nocardia* species in China are limited to a few case reports, case series, and research studies [9-12]. Particularly, there is only limited information about the species distribution and drug susceptibility of *Nocardia* [12]. Thus, the present study was designed to identify *Nocardia* species using 16SrRNA and mass spectrometry (MS) in two tertiary hospitals in China and to investigate the species distribution, clinical manifestations, microbiological characteristics, and antimicrobial susceptibility of the *Nocardia* species. In addition, we retrospectively analyzed the therapeutic effects and prognosis of patients with *Nocardia* infection.

## MATERIALS AND METHODS

### Bacterial isolates

This retrospective study was conducted at two tertiary hospitals in China, namely, the Affiliated Hospital of Jining Medical University and the Shengli Oilfield Central Hospital. A total of 27 patients with culture-proven *Nocardia* infection from January 2017 through December 2019 were included in this analysis. *Nocardia* infection was defined as a positive culture in the presence of clinical and/or radiological features of infection. Ethics approvals were obtained from the participating centers. Clinical specimens, including sputum, bronchoalveolar lavage fluid (BALF), alveolar pus, and puncture fluid were incubated on the Columbia blood agar plates at 35°C for 3–7 days. The bacterial isolates were then stored at -80°C until used. The demographic characteristics, including age, years and diseases history, as well as clinical features, laboratory tests, therapeutic regimen and outcome of included patients, were retrospectively reviewed from the medical records.

### Antimicrobial susceptibility tests

Antimicrobial susceptibility was determined using the standard Broth microdilution method following the recommendations of the Clinical and Laboratory Standards Institute (CLSI, 24^th^ edition) [13]. Antibiotics were selected according to the first-line and second-line drugs used for *Nocardia* recommended in CLSI M24-2A, including amikacin (AMK), linezolid (LNZ), trimethoprim/sulfamethoxazole (TMP-SMX), ceftriaxone (CRO), ceftazidime (CAZ), cefepime (FEP), imipenem (IPM), tobramycin (TOB), moxifloxacin (MOX), ciprofloxacin (CIP), clarithromycin (CLA), amoxicillin-clavulanic acid (AMC), and minocycline (MIN). The antibiotic susceptibility was judged as susceptible, intermediate, or resistant based on the breakpoints recommended in CLSI M24-2A. *Escherichia coli* ATCC 25922 and *Staphylococcus aureus* ATCC 25923 were used as controls.

### Identification of *Nocardia* species

Initial presumptive identification was performed according to the colony morphology on solid medium, Gram stain appearance, acid-fast staining and modified acid-fast staining, following the standard protocol. White colonies on culture plates, branching Gram-positive bacilli, positive acid-fast staining, and positive partial acid-fast staining were identified as *Nocardia* species.

*Nocardia* species were further verified by MS identification using an automated VITEK-MS system (BioMerieux, Marcy l’Etoile, France). Briefly, the bacteria isolates were mixed with 0.5 mm glass beads containing 70% ethanol (500 μL) in a pre-sterilized centrifuge tube and incubated at room temperature for 10 min. Then, the suspension was transferred into a 2.0 mL tube and centrifuged at 14,000 × g for 2 min. After ethanol was removed, the bacteria isolates were resuspended with 70% formic acid (10 μL) followed by acetonitrile (10 μL) for protein extraction. Next, the suspension was centrifuged at 14,000 × g for 2 min again and 1 μL supernatant was transferred onto target slide. After drying, 1 μL α-cyano-4-hydroxycinnamic acid (CHCA) matrix was added to complete the process. Results were analyzed using the VITEK-MS system with knowledgebase 3.0.

Bacterial genotypic identification of *Nocardia* species was further performed by 16S rRNA gene polymerase chain reaction (PCR) analysis. DNA was prepared using Bacterial Genomic DNA Extraction Kit according to the manufacturer’s instructions (TIANGEN, Beijing, China). The primer sequences of 16S rRNA used were as follows: forward primer (5´-AGAGTTTGATCCTGGCTCAG-3´) and reverse primer (5´-GGTTACCTTGTTACGACTT-3´). PCR reactions were conducted with a total reaction volume of 25 μL per well containing Phanta Max Master Mix (12.5 μL, Vazyme, Nanjing, China), forward and reverse primer (each 0.5 μL), DNA template (2.5 μL), and RNase-free water (9 μL). Amplification was performed using Veriti Thermal Cycler (Applied Biosystems, USA) under the following conditions: 95°C for 2 min, followed by 30 cycles of 95°C for 30 s, 55°C for 30 s, and 72°C for 30 s. PCR product identity was confirmed by BLAST searches of the GenBank database (https://blast.ncbi.nlm.nih.gov/Blast.cgi). Species identification results of 16S rRNA with at least 99.0% similarity were regarded as positive.

### Statistical analysis

All statistical analyses were performed by IBM SPSS Statistics for Windows, Version 22.0. (IBM Corp., Armonk, NY, USA). Quantitative data were presented by mean ± standard deviation (SD). Qualitative data were described by number or percentage.

## RESULTS

### Demographic characteristics

A total of 27 *Nocardia* isolates were identified in two centers in China during January 2017–December 2019. The demographic characteristics of patients are summarized in Table 1. There were 12 males and 15 females with a mean age (± SD) of 60.11 ± 14.94 years (median, 60 years and range, 24–87 years) identified as culture-positive *Nocardia* infection. The majority of patients (25/27, 92.6%) showed at least one underlying disease, including type 2 diabetes mellitus (7 cases), hypertension (6 cases), chronic obstructive pulmonary disease (COPD, 6 cases), bronchiectasis (5 cases), rheumatic immune diseases (5 cases), heart failure (2 cases), renal insufficiency (2 cases), malignant tumor (2 cases), and cerebral infarction (1 case).

**TABLE 1:**
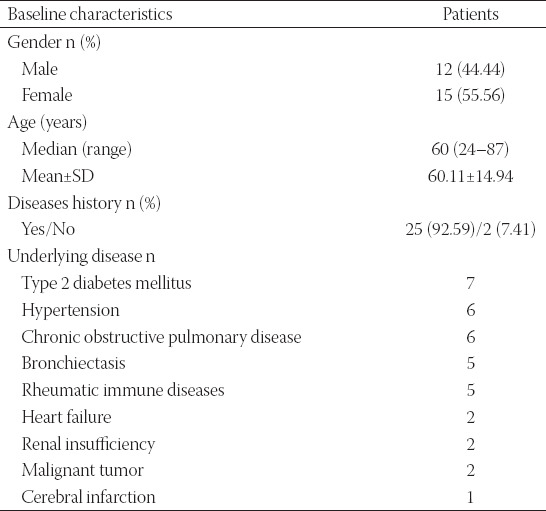
Baseline characteristics of included patients

### Phenotype identification of *Nocardia* species

**FIGURE 1:**
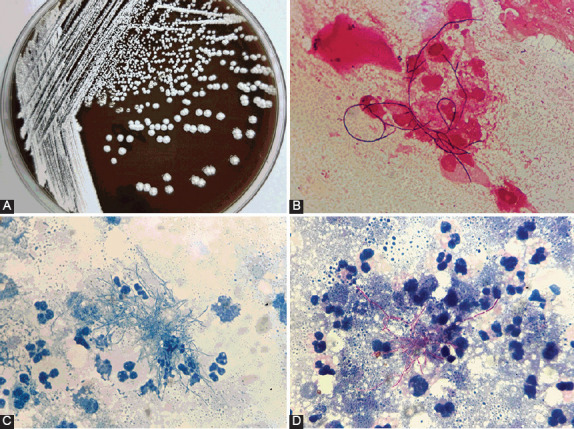
Phenotype identification of *Nocardia* species. (A) Colonies of *Nocardia* cultured on blood agar plates for 5 days and appearing as white, yellow, or orange colonies on culture plates. (B) Clinical isolates with branching Gram-positive bacilli (magnification, ×100). (C) Clinical isolates with negative acid-fast staining (magnification, ×100). (D) Clinical isolates with positive partial modified acid-fast staining (magnification, ×100).

Phenotype identification of *Nocardia* species was performed by colony morphology, Gram staining, acid-fast staining, and modified acid-fast staining. Bacterial cultures showed that *Nocardia* species were aerobic, but with a slow reproduction rate. It generally takes 3–5 days to form visible colonies and present as white, yellow, or orange colonies on culture plates (Figure 1A). Clinical isolates with branching Gram-positive bacilli (Figure 1B), negative acid-fast staining (Figure 1C), and positive partial acid-fast staining (Figure 1D) were identified as *Nocardia* species.

### Molecular identification and distribution of *Nocardia* species

**FIGURE 2:**
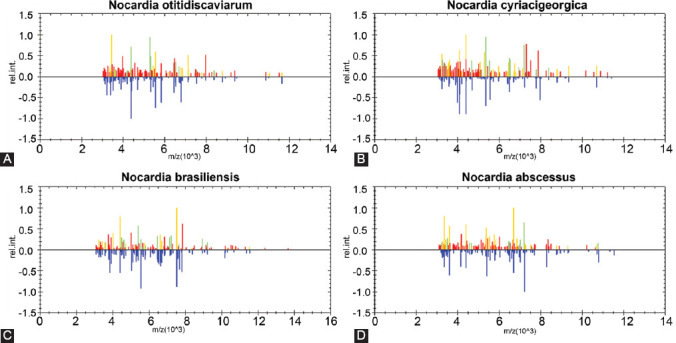
*Nocardia* isolates were identified by mass spectrometry, 7 species and 1 genus were confirmed. Representative mass spectrometry identification results of *Nocardia* species are shown: (A) *Nocardia otitidiscaviarum*; (B) *Nocardia cyriacigeorgica*; (C) *Nocardia brasiliensis*; and (D) *Nocardia abscessus*.

The 27 *Nocardia* isolates were firstly identified using the automated VITEK-MS system. Among the 27 *Nocardia* isolates, 7 species and 1 genus were confirmed. The representative MS identification results are shown in Figure 2. *N. otitidiscaviarum* was the most common species, accounting for 40.7% (11/27), followed by *N. cyriacigeorgica* (25.9%, 7/27), *N. brasiliensis* (11.1%, 3/27), *N. asteroides* (7.4%, 2/27), *N. wallacei* (3.7%, 1/27), *N. farcinica* (3.7%, 1/27), and *N. abscessus* (3.7%, 1/27). One *Nocardia* isolate has not been identified as a species by MS identification.

The *Nocardia* isolates were further confirmed by 16S rRNA PCR sequencing. A total of 7 species were identified, which was generally consistent with the results of MS identification, including *N. otitidiscaviarum* (40.7%,11/27), followed by *N. cyriacigeorgica* (25.9%, 7/27), *N. brasiliensis* (11.1%, 3/27), *N. asteroids* (7.4%, 2/27), *N. wallacei* (7.4%, 2/27), *N. farcinica* (3.7%, 1/27), and *N. abscessus* (3.7%, 1/27), respectively.

### Antimicrobial susceptibility

**FIGURE 3:**
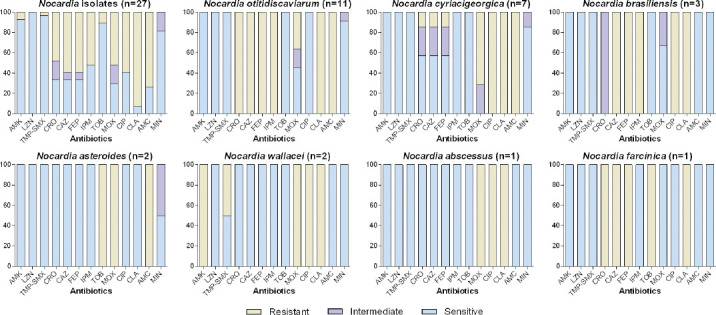
Antimicrobial susceptibility of *Nocardia* species was determined using the standard Broth microdilution method. Antimicrobial susceptibility results of 7 *Nocardia* species showed that different *Nocardia* species had different antimicrobial susceptibility profiles, but all of the isolates were LNZ susceptible. AMK: Amikacin; LNZ: Linezolid; TMP-SMX: Trimethoprim/sulfamethoxazole; CRO: Ceftriaxone; CAZ: Ceftazidime; FEP: Cefepime; IPM: Imipenem; TOB: Tobramycin; MOX: Moxifloxacin; CIP: Ciprofloxacin; CLA: Clarithromycin; AMC: Amoxicillin-clavulanic acid; MIN: Minocycline.

The susceptibility to 13 kinds of commonly-used antibiotics for *Nocardia* species is shown in Figure 3. *Nocardia* isolates showed a high sensitivity rate to AMK (92.59%), LNZ (100%), TMP-SMX (96.30%), TOB (88.89%), and MIN (81.48%). Meanwhile, *Nocardia* isolates were highly resistant to CAZ (69.26%), FEP (69.26%), CLA (92.59%), and AMC (74.07%). Notably, all of the isolates were LNZ susceptible. With regard to each *Nocardia* isolate, different *Nocardia* species had different antimicrobial susceptibility profiles, as shown in Figure 3. For example, all *N. otitidiscaviarum* isolates were highly sensitive to AMK, LNZ, TMP-SMX, TOB, CIP and MIN, while resistant to CRO, CAZ, FEP, IMP, CLA, and AMC. *N. cyriacigeorgica* isolates were sensitive to AMK, LNZ, TMP-SMX, IMP, TOB and MIN, while resistant to MOX, CIP, CLA and AMC.

### Clinical characteristics

Clinical characteristics of patients are summarized in Table 2. Patients with culture-proven *Nocardia* infection were distributed in various departments of our hospital but mostly in the Department of Respiratory Medicine (15/27, 55.56%). Besides, patients were distributed in the intensive care unit and the departments of rheumatology and immunology, orthopedics, stomatology, thoracic surgery, infection, vascular surgery, and urology. Among the 27 *Nocardia* isolates, 12 (44.44%) isolates were collected from sputum, 5 (18.52%) from BALF, 5 (18.52%) from alveolar pus, 3 (11.11%) from puncture fluid, and 2 (7.41%) were from the bronchial brush. At the time of diagnosis, high-sensitivity C-reactive protein (hs-CRP) and procalcitonin (PCT) assay was performed in 27 patients. The results showed that 21 patients had a positive CRP finding (CRP ≥8 mg/L) and 6 patients had a positive PCT finding (PCT ≥0.5 ng/mL). In addition, one patient was hemoculture positive. Lung was the most involved body site (19/27, 70.37%), followed by skin (2/27, 7.41%), pleural cavity (2/27, 7.41%), kidney (1/27, 3.70%), joint (1/27, 3.70%), maxillofacial region (1/27, 3.70%), and soft tissue (1/27, 3.70%).

**TABLE 2:**
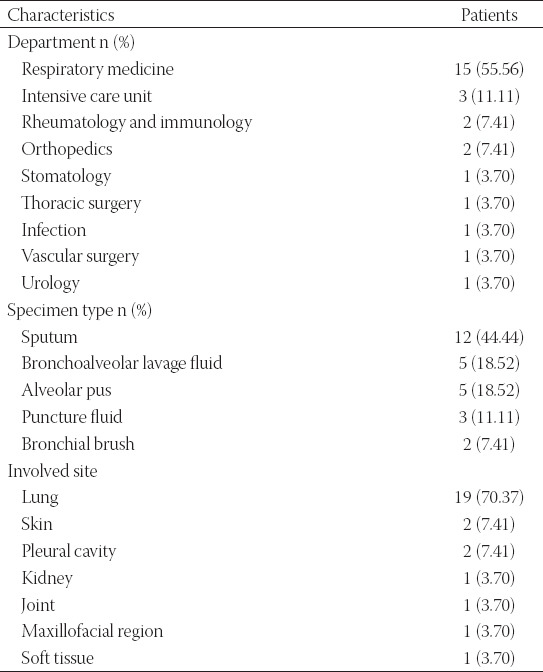
Clinical characteristics of included patients

### Treatment and outcome

Treatment information was available for all included patients. Among them, 2 patients were administrated with TMP-SMX monotherapy and 24 were administrated with the combination of TMP-SMX and other antibacterial agents such as MIN, AMK, etimicin sulfate, CIP, LNZ, and MOX. One patient was administrated with combination therapy of LNZ and cephalosporins because of allergy to sulfonamides. The treatment duration in surviving patients ranged from 7 days to 6 months. The duration of treatment in the majority of patients (81.48%) was 3 months. During treatment, no patient had abnormal blood test results nor the dysfunction of liver and kidney. Two patients experienced gastrointestinal discomfort and were relieved after omeprazole administration. Another patient had leukocytopenia and received treatment with Diyushengbai tablet. After treatment, a total of 24 patients achieved improvement and were discharged. Two deaths were reported during the treatment period. Another patient repeatedly experienced *Nocardia* infection and had been hospitalized 3 times for *Nocardia* infection of the joint until December 31, 2019.

## DISCUSSION

Previous reports indicated an increasing trend of *Nocardia* infection over the past decade [4]. In addition, *Nocardia* species are distributed differently across various geographic regions. Thus, it is necessary to investigate the epidemiology, clinical characteristics, and antimicrobial susceptibility of *Nocardia* species in different geographic regions, especially in China, a region with various geographical and climatic characteristics. Here, a total of 27 *Nocardia* isolates were obtained from 27 individuals between 2017 and 2019 at two centers in Shandong province, China. In previous studies, Yang et al. reported 40 cases with nocardiosis in a 14-year observational study between 2000 and 2013 [14] and Huang et al. analyzed 53 cases over 9 years from 2009 to 2017 [12]. Based on this evidence, we speculated that *Nocardia* infection occurred more frequently in our two centers over the past 3 years, in accordance with the overall epidemiological trends of *Nocardia* infection.

To date, more than 80 species of *Nocardia* have been described and 50 species have been identified to be human pathogens [8,12]. Nevertheless, only a few species can be reliably identified by the traditional biochemical methods, including *N. farcinica*, *N. brasiliensis*, *N. asteroides* and *N. pseudobrasiliensis* [7]. In general, the traditional biochemical methods are very time-consuming, resulting in a delayed diagnosis of infection. In recent years, 16S rRNA gene sequencing technique has become a reliable method for *Nocardia* identification [15]. However, the high cost and lack of standardization have been the major obstacle for the application of 16S rRNA gene sequencing in China. Notably, matrix-assisted laser desorption/ionization time-of-flight (MALDI-TOF) MS has emerged as an alternative method in the routine laboratory identification of *Nocardia* [16] and also has been widely applied in a large number of tertiary general hospitals in China [17]. In our study, we performed both the sequencing of 16S rRNA gene and MALDI-TOF MS to identify *Nocardia* species and obtained consistent identification results. Only one strain of *N. wallacei* was not identified to the specific species level by MS and whether this was due to technical operation or the limitations of MS remains to be further confirmed. Overall, MS has the potential to be a reliable technique for the identification of *Nocardia* species, according to the experience at our centers.

In the present study, a total of 7 *Nocardia* species were identified, of which *N. otitidiscaviarum* was the most frequently isolated, followed by *N. cyriacigeorgica*. Inconsistently, in previous studies, the predominant *Nocardia* species varied in different regions; namely, *N. nova*, *N. abscessus* and *N. cyriacigeorgica* were, respectively, the most predominant species in the United States [18], Italy [19], and Spain [20]. Even in China, the distributional features of *Nocardia* species varied in different regions [12,21-23], indicating that the distribution of *Nocardia* species might be geographically-related.

Treatment for *Nocardia* infection according to the susceptibility of the isolated species is essential whenever possible, thereby, understanding the species-specific antimicrobial susceptibility patterns is very important. We reported the antimicrobial susceptibility patterns of the first-line and second-line drugs used for *Nocardia* infection. Here, the antimicrobial susceptibility profile was largely consistent with the results of the antibiograms available in the literature [19,22]. We found that the *Nocardia* species had good susceptibility to LNZ, TMP-SMX, and AMK in the present study. Furthermore, our isolates showed varying susceptibilities to different antibiotics. However, each species had a limited number of strains, which may not be enough to accurately represent the antimicrobial susceptibility. Thus, further studies with a larger sample size are still needed to confirm the antibiograms from our region.

In general, the majority of patients with *Nocardia* infection have a certain degree of immune deficiency, particularly patients with underlying diseases or immunosuppressive treatment. In the present study, approximately 93% of patients had at least one known underlying disease responsible for immune deficiency, which was consistent with the previous data [4,23]. The most common underlying diseases were type 2 diabetes mellitus, hypertension, and COPD. It is reported that impaired local pulmonary deficiency caused by COPD predispose to pulmonary nocardiosis, particularly in patients requiring long-term corticosteroid treatment [24]. Among the 27 included cases, 2 (7.41%) cases showed no evidence of underlying diseases. This data ranges from 10% to 25% in other reports [4]. Thus, *Nocardia* infection can occur both in immunocompetent and immunocompromised hosts.

With regard to the site of involvement, the pulmonary region was the most commonly involved body site of the patients in our two centers, which is consistent with the previous findings [14,19]. In addition, a literature review that included more than 1000 patients also reported that the pulmonary apparatus was the most commonly involved site [25]. In fact, it is well known that, because of its transmission route (direct inhalation or inoculation), *Nocardia* infections are mainly localized in the lower respiratory tract and the soft tissue. Nevertheless, nocardiosis is mostly an opportunistic infection leading to severe, life-threatening disseminated infections. Occasionally, some patients with disseminated infection may show the brain abscess, brain involvement, or neurological deficits [26,27]. No brain involvement was reported in our retrospective analysis, however, this may be due to the small sample size and the possibility should not be discounted. These findings suggest that either lung or brain computed tomography should be performed routinely in patients with *Nocardia* infections.

The laboratory test results were available in all 27 patients and showed that most patients had positive hs-CRP and approximately 20% of patients had positive PCT. Similarly, Guo et al. reported that most patients with nocardiosis had an elevated CRP level, whereas PCT levels were normal or slightly elevated [28]. These results suggest some typical indicators of *Nocardia* infection and increasing PCT levels may indicate a severe infection.

At present, antibiotic treatment is a major therapeutic strategy for *Nocardia* infection and, among them, TMP-SMX has been preferred and used as the first-line therapy [29]. For some patients with a serious infection and long duration, TMP-SMX-based combination therapy is recommended in clinical setting [30]. Unfortunately, an optimal therapeutic strategy has not been well defined in clinical practice. In the present study, TMP-SMX was the most commonly administered treatment and is still considered as a primary treatment option at our centers. The majority of our patients received TMP-SMX-based combination therapy. Recently, LNZ showed favorable clinical effectiveness in *Nocardia* infections, and it has the potential to be a useful alternative therapeutic agent due to its oral availability and activity against most *Nocardia* species [31]. In our study, since one patient was allergic to SMX, the combination therapy of LNZ and cephalosporins was administrated. After the corresponding therapy, nearly 90% of our patients achieved improvement and were discharged. The mortality rates associated with *Nocardia* infection reported in the literature range from 26% to 63% [32]. The mortality rate was 7.41% in our study, which is at a relatively low level. It is demonstrated that the mortality rate depends on the underlying disease, degree of immunosuppression, and disseminated form of *Nocardia* infection [33]. The clinical course of one patient in the present study, who experienced recurrent infection during treatment, was very complicated. This case suggested that follow-up treatment is necessary and may protect against relapses and dissemination.

## CONCLUSION

The present study retrospectively analyzed 27 non-repetitive clinical *Nocardia* isolates from two centers in Shandong, China. We found that the clinical manifestations of *Nocardia* infection and antimicrobial susceptibility profiles varied with different *Nocardia* species. Besides, MALDI-TOF MS was proved as a rapid and accurate identification for the *Nocardia* species. Our findings suggest the necessity of accurate species identification and confirmation of antimicrobial susceptibility patterns in the diagnosis and the choice of antibiotic treatment.
